# An image-based approach for the estimation of arterial local stiffness *in vivo*

**DOI:** 10.3389/fbioe.2023.1096196

**Published:** 2023-01-30

**Authors:** Simona Celi, Emanuele Gasparotti, Katia Capellini, Francesco Bardi, Martino Andrea Scarpolini, Carlo Cavaliere, Filippo Cademartiri, Emanuele Vignali

**Affiliations:** ^1^ BioCardioLab, UOC Bioingegneria, Fondazione Toscana G Monasterio, Massa, Italy; ^2^ Mines Saint-Etienne, Universit’e de Lyon, INSERM, SaInBioSE U1059, Lyon, France; ^3^ Dipartimento di Ingegneria Industriale, Università “Tor Vergata”, Roma, Italy; ^4^ Dipartimento di Radiologia, IRCCS SynLab SDN, Napoli, Italy; ^5^ Dipartimento Immagini, Fondazione Toscana G. Monasterio, Pisa, Italy

**Keywords:** *in vivo* arterial stiffness, tissue mechanics, ECG-gated CT images, mesh-morphing, inverse engineering

## Abstract

The analysis of mechanobiology of arterial tissues remains an important topic of research for cardiovascular pathologies evaluation. In the current state of the art, the gold standard to characterize the tissue mechanical behavior is represented by experimental tests, requiring the harvesting of *ex-vivo* specimens. In recent years though, image-based techniques for the *in vivo* estimation of arterial tissue stiffness were presented. The aim of this study is to define a new approach to provide local distribution of arterial stiffness, estimated as the linearized Young’s Modulus, based on the knowledge of *in vivo* patient-specific imaging data. In particular, the strain and stress are estimated with sectional contour length ratios and a Laplace hypothesis/inverse engineering approach, respectively, and then used to calculate the Young’s Modulus. After describing the method, this was validated by using a set of Finite Element simulations as input. In particular, idealized cylinder and elbow shapes plus a single patient-specific geometry were simulated. Different stiffness distributions were tested for the simulated patient-specific case. After the validation from Finite Element data, the method was then applied to patient-specific ECG-gated Computed Tomography data by also introducing a mesh morphing approach to map the aortic surface along the cardiac phases. The validation process revealed satisfactory results. In the simulated patient-specific case, root mean square percentage errors below 10% for the homogeneous distribution and below 20% for proximal/distal distribution of stiffness. The method was then successfully used on the three ECG-gated patient-specific cases. The resulting distributions of stiffness exhibited significant heterogeneity, nevertheless the resulting Young’s moduli were always contained within the 1–3 MPa range, which is in line with literature.

## 1 Introduction

The analysis of arterial tissue remains a pivotal topic of research in the field of cardiovascular pathologies. It was well established that a plethora of cardiovascular diseases find their origin within the mechanics and the biology of the vessel tissues (Humphrey and Schwartz. (2021)). Attention was focused on both large and small vessels including different types of pathologies like dissections, stenosis/atherosclerotic arteries and aneurysms (Celi et al. (2013); Gültekin et al. (2019); Vignali et al. (2020)). An aneurysm is defined as a local dilatation in the aortic wall, that is usually asymptomatic up to the sudden rupture which may be linked with patient’s death (Ramanath et al. (2009)). The current clinical practice is to define a critical aortic size criterion to determine the necessity of surgical implantation. The aneurysm pathology remains an open clinical challenge and it still requires a deep insight in terms of formation and progression mechanisms. Different studies reported that the critical state of an aneurysm case arises from the status of the tissue biomechanics and its degradation (Vignali et al. (2020); Vignali et al. (2021c)). The usage of the mechanical analysis principle could ideally provide an improved understanding of the aneurysm nature and, in general, of the arterial behavior under given pathological conditions.

Following this analysis principle, different groups have provided mechanical insights concerning the arterial tissues. In the current state of the art, various experimental testing procedures have been proposed, like uniaxial/biaxial traction tests (Vignali et al. (2021a); Peña et al. (2015)) and bulge inflation approaches (Duprey et al. (2016)). It is also worth noting that several studies were focused on the investigation of correlation of biological and mechanical features of the arterial tissue (Vignali et al. (2020; 2021c)), given their important link. This literature field presents a shared flaw, which is the necessity of *ex-vivo* tissue samples to be tested. Given this, the mechanical analysis is necessarily limited to post-operative cases, in which the surgical procedure has already been performed. Consequently, it is impossible to have a direct mechanical characterization of the arterial tissue without an invasive procedure.

Obtaining mechanical features of the arterial tissue non-invasively still represents an open research topic. Nevertheless, research efforts towards this direction have been made recently. Several image-based approaches have already been explored to estimate mechanical properties of soft tissues in general (Fanni et al. (2020); Di Lascio et al. (2014)). These *in vivo* estimation methods are made possible thanks to the recent advances in terms of clinical imaging (Celi et al. (2017)), which allow high-resolution reconstruction of cardiovascular structures at different cardiac phases. The dynamic nature of imaging techniques like ECG-gated Computed Tomography (CT), echography and 4D Magnetic Resonance Imaging (4D-MRI) opens the possibility to reconstruct the displacement fields of cardiovascular structures.

The main focus of non-invasive mechanical analysis resides mainly in strain estimation on vessels like the ascending aorta section. The reported *in vivo* strain evaluation techniques are usually based on mapping algorithms aimed at reconstructing the aortic kinematics along the cardiac cycle. Among the different mapping techniques, iterative registration approaches (Liu et al. (2019b); Narayanan et al. (2021)), projections along the normal of the aortic surface (Pasta et al. (2017)) and centerline-based decompositions with parametric templates (Farzaneh et al. (2019b; Farzaneh et al. (2019a)) were proposed. Beyond the knowledge of aortic strain, stress is still required for a stiffness estimation. Nevertheless, the *in vivo* evaluation of stress remains a difficult task. For this reason, different studies were limited to strain-only analyses (Pasta et al. (2017)), or presented assumptions on the load to infer simplified stress distributions (Duprey et al. (2016); Martin et al. (2013)). Some groups proposed iterative Finite Element (FE) approaches for the direct estimation of aortic stiffness (Krishnan et al. (2015)), but the requirement for multiple numerical simulations can be computationally onerous. Other groups also proposed aortic volumetric distensibility as a surrogate for stiffness estimation (Trabelsi et al. (2018)), but the global nature of the parameter did not allow for a local estimation of the material properties.

With the current study, a new method for the strain and stiffness estimation of the aortic vessel is proposed. The method aims at providing local information, in opposition with volumetric/global approaches (Trabelsi et al. (2018); Danpinid et al. (2009)) by relying on CT data, centerline calculation and mesh-morphing based mapping. Other previous studies relied on centerline-based information (Zeinali-Davarani et al. (2011)), nevertheless their focus was more centered on the abdominal aorta district and they used the centerline to map wall thickness interpolations along the vessel. To our knowledge, this is the first approach proposing a mesh-morphing sequence to allow for the mapping of the aortic surface along the different cardiac cycle phases. The morphing of the baseline mesh on the different deformed surfaces can allow for a fast method for mapping and, consequently, it gives a great potential for local strain evaluation. Concerning the stress, a further step to go beyond the literature relying on the Laplace hypothesis (Liu et al. (2019a); Martin et al. (2013)) could be imposed by relying on inverse FE simulations.

The aim of the current work is to propose a new approach to provide local distribution of arterial stiffness, based on the knowledge of *in vivo* patient-specific imaging data. The method is based on the calculation of aortic strain on the basis of contour length ratios for each section. For the estimation of stress, two approaches were tested: an approach based on Laplace hypothesis, and an approach based on an inverse engineering FE simulation to estimate the distribution of wall tension on the aortic geometry. The entire procedure was first validated on a set of FE simulations. The two estimation methods were first compared for the validation phase. Finally, the approach was applied on three patient-specific ECG-gated CT data to estimate the local stiffness distribution *in vivo*. The results are then presented and discussed to assess the new method performances and future applications.

## 2 Materials and methods

In this section the three main steps of the entire pipeline are presented: method description (Subsection 2.1), validation (Subsection 2.2) and application (Subsection 2.3).

### 2.1 Method for non-invasive stiffness estimation

**FIGURE 1:**
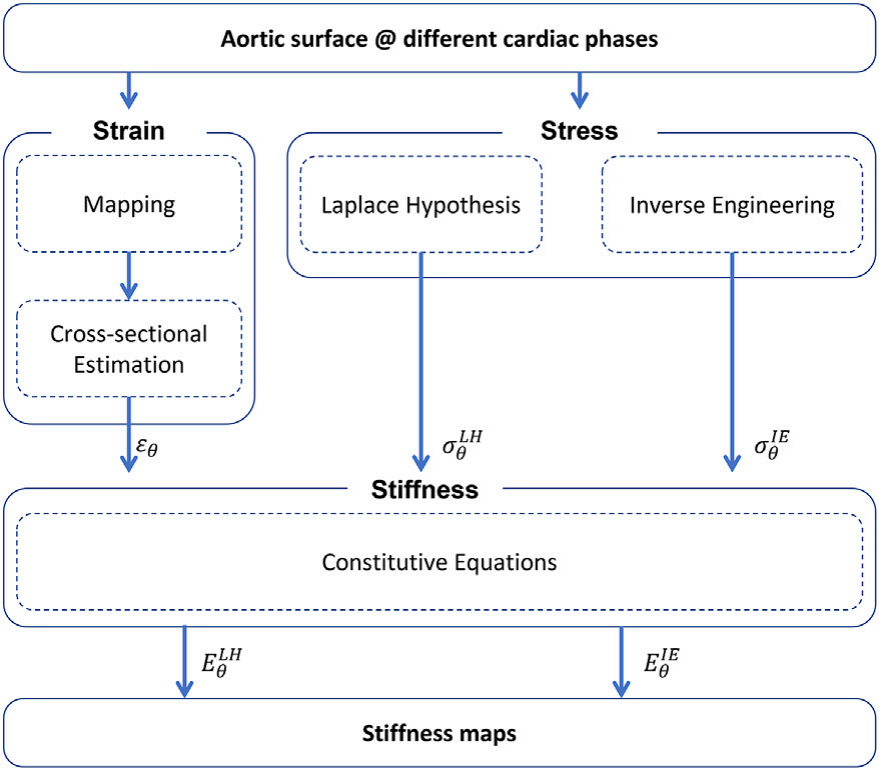
Summary of the workflow for the non-invasive stiffness estimation (*ɛ*_*θ*_—circumferential strain, $\sigma_{\theta }^{LH,IE}$ —circumferential stress estimated with Laplace Hypothesis (LH) or with inverse engineering (IE) approach, $E_{\theta }^{LH,IE}$ —circumferential stiffness estimated with Laplace Hypothesis (LH) or with inverse engineering (IE) approach.

The methods for the stiffness estimation are described in the workflow depicted in Figure 1. The workflow is divided in three main parts: strain i), stress ii) and stiffness iii) estimation.

*Strain estimation—*A mapping procedure is required first. The mapping purpose is to define a nodal mesh which can be tracked across each reconstructed aortic geometry. Each node must be mapped to represent the position of the same material point at each phase of the cardiac cycle. For this reason, it is fundamental to define a mesh for all phases with the same number of nodes and connectivity. To achieve this, a mesh morphing approach based on radial-basis functions interpolation is adopted (Capellini et al. (2018); Capellini et al. (2021)). Briefly, the surfaces at each of the different cardiac phases are calculated by taking the 0% phase as the reference mesh, that is the baseline surface configuration (Σ_*dia*_). The baseline surface mesh is then morphed onto the deformed surfaces, according to radial basis functions interpolation. The source points to morph the initial mesh onto the other phases were selected on the basis of a sphere grid within the ascending aorta section. At each phase, specific sets of target points were chosen. After the procedure, the result is given by a set of deformed meshes, including the peak systolic phase (Σ_*sys*_).

**FIGURE 2:**
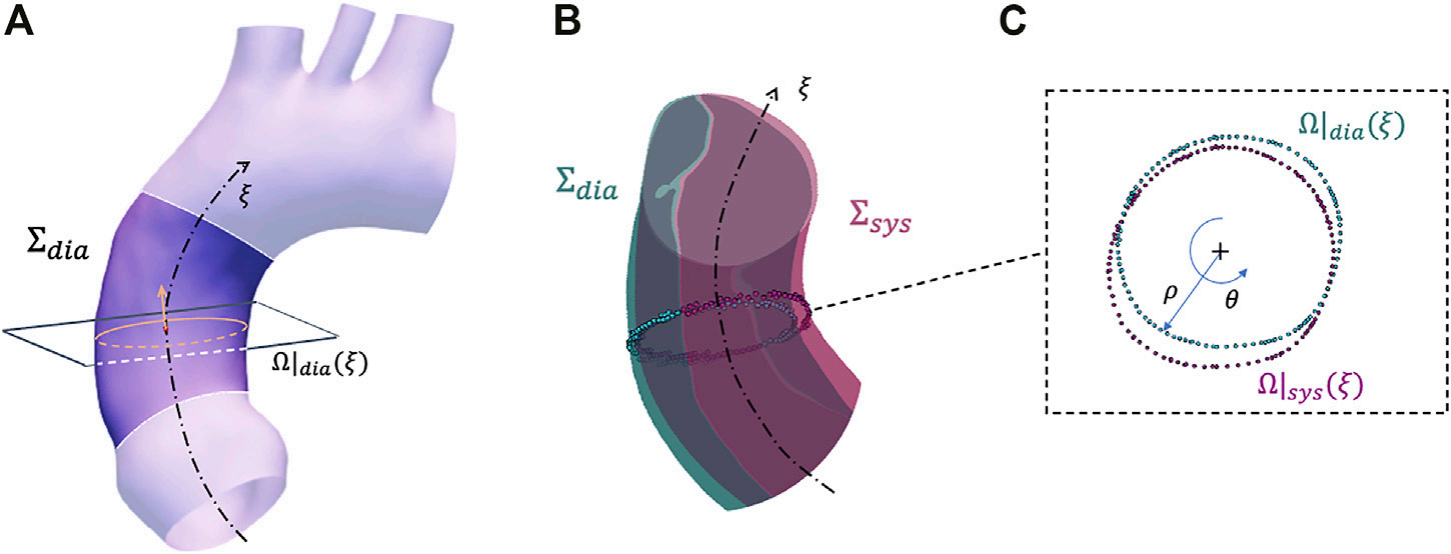
Summary of the strain estimation algorithm: example of a plane and closed contour definition on configuration Σ_*dia*_ **(A)**, corresponding closed contour definition on configuration Σ_*sys*_ **(B)** with closeup on plane with polar coordinates **(C)**.

After the mapping procedure, a specific algorithm is developed to estimate the strain from the mapped aortic surfaces in the Σ_*dia*_ and Σ_*sys*_ configurations. The algorithm is based on sectional contour length ratios and it is summarized in Figure 2. The choice of defining sectional contours to estimate the strain is motivated by the fact that the imposed mapping does not account for physiological deformations of the vessel but it is based on surface fitting optimizations (Sieger et al. (2014)). In brief, the centerline (*ξ*) of the Σ_*dia*_ configuration is evaluated first. Then, for each centerline coordinate *ξ*, a set of nodes was extracted representing a given cross section of the vessel. The cross sectional points were selected according to a distance threshold from the surface Σ_*dia*_ and a given plane defined from the centerline tangent. It is important to highlight that it is sufficient to evaluate the centerline on the baseline configuration only, as the movement of the centerline itself is already taken into account by considering the corresponding cross sectional points, thanks to nodal mapping. It is reasonable to consider that during the cardiac cycle the aorta experiences longitudinal displacement as well. In fact, the mapping provided by the morphing of the baseline surface onto the deformed surface also accounts for axial displacement.

At the *ξ* centerline coordinate, a closed contour Ω|_*dia*_(*ξ*) is defined by sorting the cross sectional points according to a polar coordinate conversion (Figure 2A). At this point, the Ω|_*dia*_(*ξ*) radial contour length is defined as:

$$L{|}_{\mathrm{dia}}\left(\xi \right)=\int_{\Omega {|}_{\mathrm{dia}}\left(\xi \right)}\rho \left(\theta \right)d\theta$$ (1)

where *ρ*(*θ*) and *θ* are the polar coordinates (radial and circumferential, respectively) defined for the cross sectional plane at the *ξ* centerline coordinate point. Thanks to the mapping procedure, it was possible to identify the corresponding cross sectional nodes for each centerline node at the Σ_*sys*_ configuration. This permitted the definition of a closed contour at the *ξ* centerline coordinate within the Σ_*sys*_ configuration (Ω|_*sys*_(*ξ*)) and, by applying Eq. 1 again, the definition of the corresponding length (*L*|_*sys*_(*ξ*)) (Figures 2B, C). As circumferential strain represents the change in the length along the aorta cross section, it is possible to define the sectional contour length ratio as:

$$\varepsilon_{\theta }\left(\xi \right)=\frac{L{|}_{\mathrm{sys}}\left(\xi \right)-L{|}_{\mathrm{dia}}\left(\xi \right)}{L{|}_{\mathrm{dia}}\left(\xi \right)}$$ (2)

where *ɛ*_*θ*_(*ξ*) is the circumferential strain at the aortic cross section identified by the centerline coordinate *ξ*. By assuming this, the obtained strain distribution results to be a function of the centerline coordinate *ξ*.

*Stress estimation—*For the evaluation of local stress distribution, two main approaches were adopted: a Laplace-hypothesis-based (LH) and an inverse-engineering-based (IE) method.

For the first method, the assumption of a thin walled surface is made for the aortic structure, with negligible curvature at the ascending section (Liu et al. (2019a)). The negligibility of curvature is checked according to the following condition (Zhang et al. (2013)):

$$m=\frac{R_{0}-r}{R_{0}-r/2}\approx 1$$ (3)

where *m* is the curvature effect factor, *R*_0_ is the centerline radius of curvature and *r* is the section radius. On the basis of this, it is safe to evaluate the circumferential stress as the wall hoop stress. By considering each cross section of the aortic centerline, the corresponding nodal circumferential stress according to the Laplace method is evaluated as:

$$\sigma_{\theta }^{LH}\left(\xi \right)=\frac{\Delta P\bar{\rho }}{\delta }$$ (4)

where $\sigma_{\theta }^{LH}\left(\xi \right)$ and *δ* are the circumferential stress and the thickness of the aortic vessel, while Δ*P* is the pressure difference between the systolic and diastolic condition and $\bar{\rho }$ is the mean radius at the centerline coordinate *ξ*. The mean radius was calculated by considering the mean radial coordinate, according to the polar coordinate system already defined for the sectional contour calculation (see Eq. 1).

For the second alternative method, an inverse engineering (IE) approach is chosen (Lu et al. (2008); Zhou et al. (2010)). It is well known that the wall tension in a pressurized membrane is equilibrium-determinate and it depends exclusively on the morphology. Briefly, a structural FE simulation is setup to evaluate the circumferential stress distribution at each node of the mapped mesh. To obtain a stress distribution, depending on the aortic morphology only, the deformed geometry in systolic configuration was loaded with an internal pressure of Δ*P*. The aorta was modeled as a membrane with a practically undeformable isotropic material (Young’s modulus (*E*) > 10 GPa). By considering each cross section of the aortic centerline, the mean circumferential stress from the inverse method at a given point for the centerline can be evaluated as:

$$\sigma_{\theta }^{IE}\left(\xi \right)=\frac{1}{N}\overset{N}{\underset{i\in \Omega {|}_{\mathrm{sys}}\left(\xi \right)}{\sum }}\sigma_{\theta }^{i}\left(\xi ,\theta_{i}\right)$$ (5)

where $\sigma_{\theta }^{IE}\left(\xi \right)$ is the nodal maximum principal stress resulting from the simulation and *N* is the number of nodes in a sector of section Ω|_*sys*_(*ξ*). By assuming this model, it is possible to account for curvature effect on stress within the aortic domain.

*Stiffness estimation*—The definition of stiffness from the evaluation of strain and stress is, at last, performed. To evaluate the stiffness, the model was assumed as linearized, given the possibility to assume small deformations occurring between diastolic and systolic phase (Vignali et al. (2021b); Roccabianca et al. (2014)). The assumption allowed for the adoption of the Hooke law as a constitutive equation to relate stress and strain. By assuming a negligible radial and longitudinal stress and by considering Eqs 2, 4, and 5, the following can be imposed to estimate the circumferential stiffness:

$$E_{\theta }^{LH}\left(\xi \right)=\frac{\sigma_{\theta }^{LH}\left(\xi \right)}{\varepsilon_{\theta }\left(\xi \right)};\;E_{\theta }^{IE}\left(\xi \right)=\frac{\sigma_{\theta }^{IE}\left(\xi \right)}{\varepsilon_{\theta }\left(\xi \right)}$$ (6)

where $E_{\theta }^{LH}$ and $E_{\theta }^{IE}$ represent the circumferential Young’s moduli evaluated according to the two different stress estimation techniques already described.

### 2.2 Numerical validation

**FIGURE 3:**
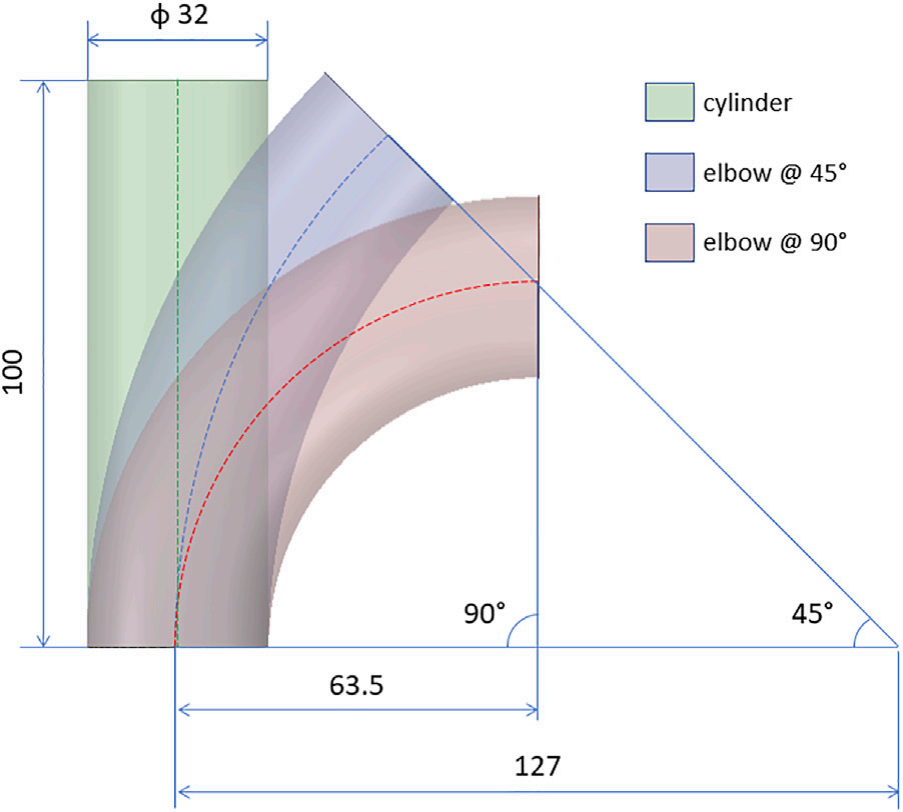
Design of the idealized geometries for the validation cases: cylinder and elbows at 45° and 90°. All quotations are given in mm.

**FIGURE 4:**
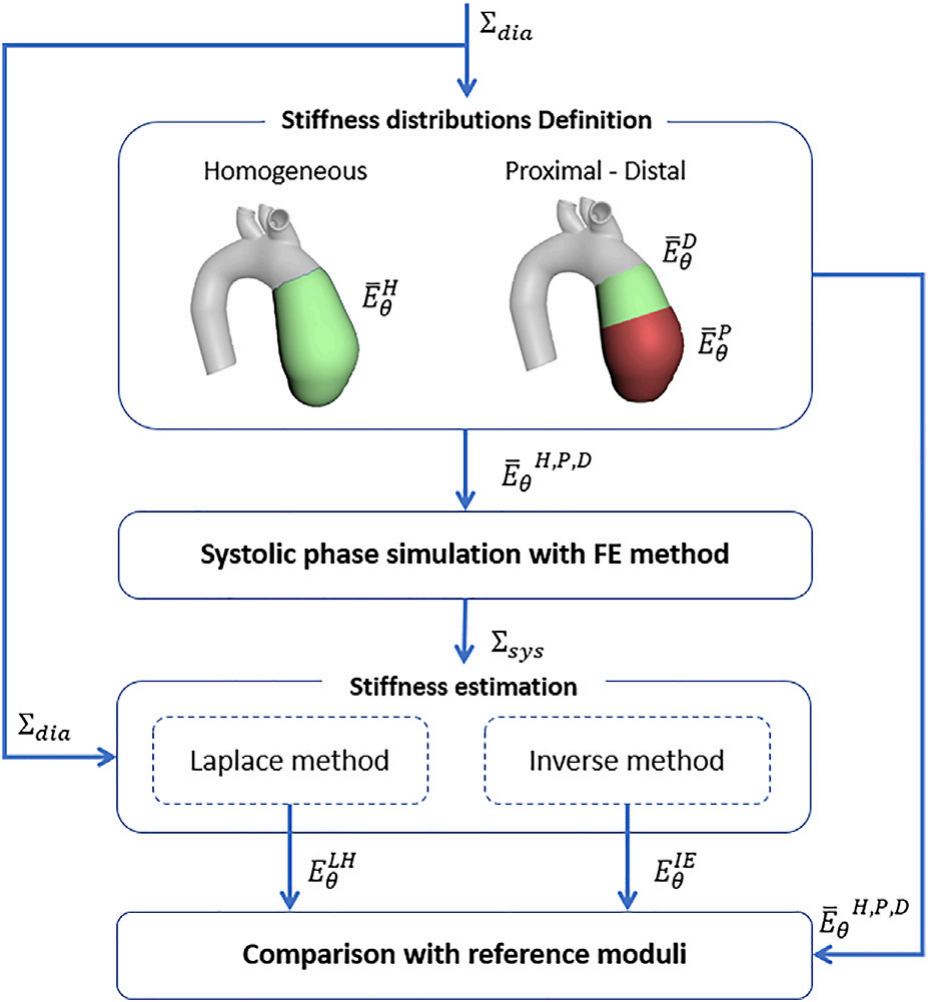
Numerical validation workflow for the stiffness estimation algorithm on the basis of FE simulations.

After defining the stiffness estimation methods, the technique was validated according to a FE approach. Firstly, idealized synthetic geometries were defined. In particular, a cylinder, a 45° and a 90° elbow geometries were defined, to assess the influence of curvature (Figure 3). All the idealized geometries were designed with a diameter of 32 mm and a length of 100 mm. In addition, a patient-specific test case was selected from a segmented aortic geometry. For all the cases, a thickness of 2 mm was assumed. The numerical workflow for the validation on the patient-specific simulated case is summarized in Figure 4. The geometry of the ascending aorta was taken from a contrast-enhanced CT dataset with ECG-gating, obtained with a 320-detector scanner (Toshiba Aquilon One, Toshiba, Japan). Thanks to the ECG-gating, the diastolic phase was selected for the segmentation. The segmentation procedure was carried out through a semi-automatic region-growing algorithm following the approach previously described in Celi et al. (2021). The validation procedure can be summarized in three main phases: material properties distribution i), systolic phase definition ii), stiffness estimation application and comparison iii). All the simulation activities were carried out within the ANSYS environment.

*Material properties distribution definition—*For the idealized geometries, a linear elastic isotropic homogeneous distribution of stiffness was assumed. A single value of Young’s modulus ( ${\bar{E}}_{\theta }^{H}$ = 0.5 MPa) was imposed. Concerning instead the simulated patient-specific case, four main cases of Young’s modulus distribution were simulated and used as validation: three linear elastic isotropic homogeneous (H) distribution with a single Young’s Modulus ( ${\bar{E}}_{\theta }^{H}$ = 0.5 MPa, 1.75 MPa, 3.0 MPa) i) and a single proximal/distal (PD) distribution with a proximal ( ${\bar{E}}_{\theta }^{P}$ = 2.0 MPa) and distal ( ${\bar{E}}_{\theta }^{D}$ = 0.5 MPa) Young’s Modulus ii) (Figure 4). In the PD distribution case, the variation from ${\bar{E}}_{\theta }^{P}$ to ${\bar{E}}_{\theta }^{D}$ was implemented with a step transition. The values were chosen in order to be contained within the physiological range of stiffness of the ascending aorta (Lin et al. (2022)). In this way, it was possible to estimate the potential of the proposed method to evaluate the possibility to recover a local distribution of stiffness. The imposed values were taken as reference for the validation of the stiffness estimation method.

*Systolic phase definition—*For the idealized and patient-specific geometries with all the considered stiffness distributions, the surface configuration in the systolic phase was simulated. In particular, static structural simulations were imposed to obtain the systolic configuration starting from the diastolic configuration, designed for the idealized cases and segmented for the patient-specific case. In particular, an internal pressure of 40 mmHg, according to the physiological pressure difference between systole and diastole, was imposed for all cases. The aortic valve plane was constrained with a fixed displacement condition, while the radial displacement was left free for the aortic arch plane. It is worth noting that for all the FE validation cases it is not necessary to consider the mapping for strain estimation (see Figure 1), as the nodes are already mapped by the structured mesh.

*Stiffness estimation application and comparison—*The diastolic and systolic surfaces resulting from the FE simulations, for both the idealized and patient-specific models with all the stiffness distributions, were set as input for the estimation method described in Subsection 2.1. Both methods based on Laplace hypothesis and inverse engineering were used to calculate $E_{\theta }^{LH}$ and $E_{\theta }^{IE}$ as summarized in Eq. 6. The resulting stiffness maps and the Relative error (*RErr*) in percentage for both LH and IE method were considered. Additionally, the stiffness distributions resulting from the simulated patient-specific validation cases were evaluated along the normalized centerline coordinate *ξ* of both $E_{\theta }^{LH}$ and $E_{\theta }^{IE}$ . The average root mean square percentage error (RMSPE) along the centerline, relative to the reference values of Young’s moduli imposed at simulation level, was considered for all validation cases.

### 2.3 Patient-specific cases

After evaluating the performances of the methods on FE validation cases, the estimation technique was imposed by using patient-specific data as input. The analysis procedure can be summarized in three main phases: image acquisition and processing i), systolic phase definition ii), stiffness estimation application iii).

*Image acquisition and processing—*Three patient-specific aortic morphology were reconstructed from *in vivo* data. In particular, three datasets of 5-phase ECG-gated CT images were acquired. The following percentages of cardiac cycle phases are considered for the analysis: 0%, 20%, 40%, 60% and 80%. The cases selected were three males (25, 89 and 64 y.o.) with tricuspid aortic valve conformation. The CT images were obtained with a 320-detector scanner (Toshiba Aquilon One, Toshiba, Japan) by adopting a iodinated contrast medium. For each phase of the three cases, the ascending aorta morphology was reconstructed according to a semi-automated segmentation algorithm. For each of the three patient-specific cases, the Signal-to-Noise-Ratio (SNR) was calculated by considering a ROI within the ascending aorta section and by calculating the ratio between the pixel mean and standard deviation.

Together with the morphologies, the systemic pressure range was acquired, according to the corresponding clinical record, for each analyzed case. In particular pressure ranges of 82–120 mmHg (Case 1), 78–122 mmHg (Case 2) and 80–124 mmHg (Case 3) were reported. It is important to notice that the pressure ranges (Δ*P*_1_ = 38 mmHg, Δ*P*_2_ = 44 mmHg, Δ*P*_3_ = 44 mmHg) for the chosen cases can be considered as physiological. This aspect confirms the possibility to assume small deformations occurring between diastole and systole and to linearize the material response (Gundiah et al. (2008); Vignali et al. (2021b)).

*Systolic peak phase definition—*The resulting aortic surfaces from the image segmentation phase are adopted and used as input for the procedure described in Subsection 2.1. In brief, the segmented surfaces from clinical data were mapped according to the already described morphing technique and the baseline centerline was calculated. Then, the corresponding circumferential strain maps at each phase recorded by the ECG-gating process were calculated. Each strain map was calculated considering the 0% phase as the Σ_*dia*_ baseline reference. The Σ_*sys*_ phase was then individuated. To choose the Σ_*sys*_ phase among the different cardiac cycle phases form the ECG-gating, the different strain maps were analysed first. By assuming that the configuration revealing the maximum strain was the one associated with the most pressure difference, Σ_*sys*_ was chosen by selecting the phase revealing the highest circumferential strain for each case.

*Stiffness estimation application—*After the selection of the Σ_*sys*_, the procedure for stiffness estimation was carried out, according to the methods already described in Subsection 2.1. The stiffness maps were evaluated on the ascending aorta section of the patient-specific cases by considering the baseline surface and the systolic phase only, as selected in the previous step. Additionally, the resulting stiffness maps and distributions along the centerline coordinate *ξ* were evaluated for each case.

## 3 Results

**FIGURE 5:**
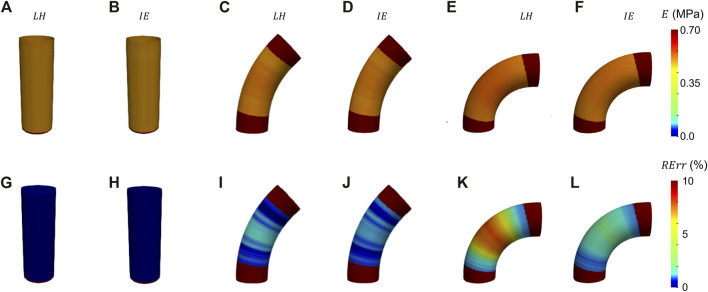
Young’s modulus maps of FE validation for idealized geometries **(A–F)** with corresponding relative errors **(G−I)**. The maps are presented for both LH **(A, G, C, I, E, K)** and IE (**B, H, D, J, F, L**) methods.

The results from the FE validation procedure are presented first. Concerning the simplified geometries, the curvature effect factor is reported first. According to Eq. 3, values of *m* = 1, m = 0.93 and *m* = 0.86 were calculated for the cylinder and the elbows at 45° and 90°, respectively. The results in terms of Young’s modulus maps for cylinder and elbows geometries are presented in Figures 5A–F. The relative error maps, calculated according to the reference value imposed at simulation level, are reported as well (Figures 5G–L). The corresponding RMSPE values for all the idealized geometries are reported in Table 1.

**TABLE 1:** Table summarizing the Young’s modulus RMSPE, relative to the reference values for all validation cases, including idealized and patient-specific.

Validation case		RMSPE for LH (%)	RMSPE for IE (%)
Idealized	Cylinder	1.4	0.6
	Elbow at 45°	2.2	1.7
	Elbow at 90°	6.0	4.4
Patient-specific	${\bar{E}}_{\theta }^{H}$ = 0.50 MPa	10.1	9.6
	${\bar{E}}_{\theta }^{H}$ = 1.75 MPa	9.9	9.7
	${\bar{E}}_{\theta }^{H}$ = 3.00 MPa	9.4	8.5
	Proximal/Distal	16.3	16.1

**FIGURE 6:**
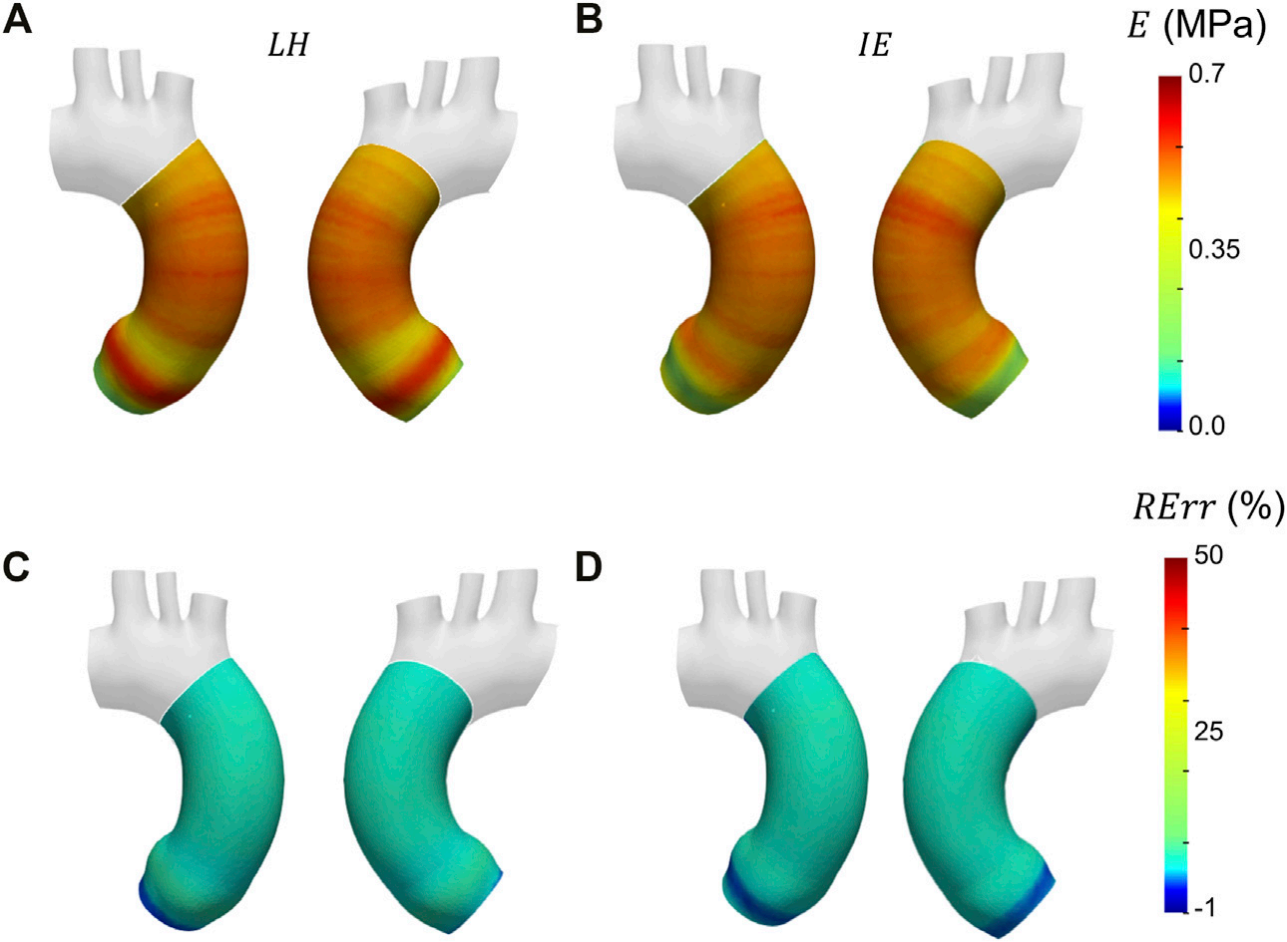
Young’s modulus maps of FE validation for patient-specific geometry with homogeneous distribution with ${\bar{E}}_{\theta }^{H}=$ 0.5 MPa **(A, B)** with corresponding relative errors **(C, D)**. Both Laplace-hypothesis-based $\left(E_{\theta }^{LH}\right)$ **(A, C)** and inverse-engineering-based $\left(E_{\theta }^{IE}\right)$ **(B, D)** results are presented.

**FIGURE 7:**
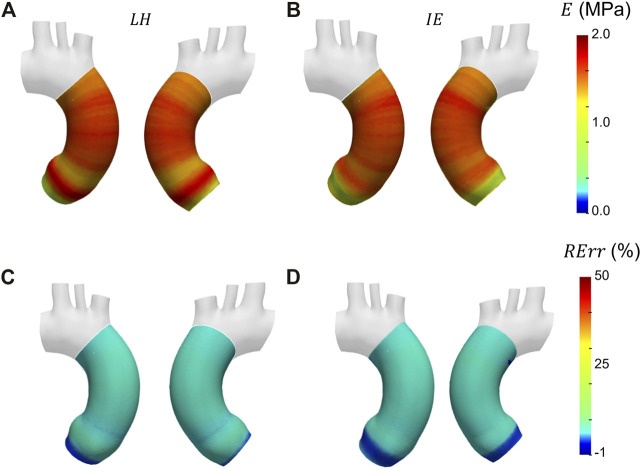
Young’s modulus maps of FE validation for patient-specific geometry with homogeneous distribution with ${\bar{E}}_{\theta }^{H}=$ 1.75 MPa **(A, B)** with corresponding relative errors **(C, D)**. Both Laplace-hypothesis-based $\left(E_{\theta }^{LH}\right)$ **(A, C)** and inverse-engineering-based $\left(E_{\theta }^{IE}\right)$ **(B, D)** results are presented.

**FIGURE 8:**
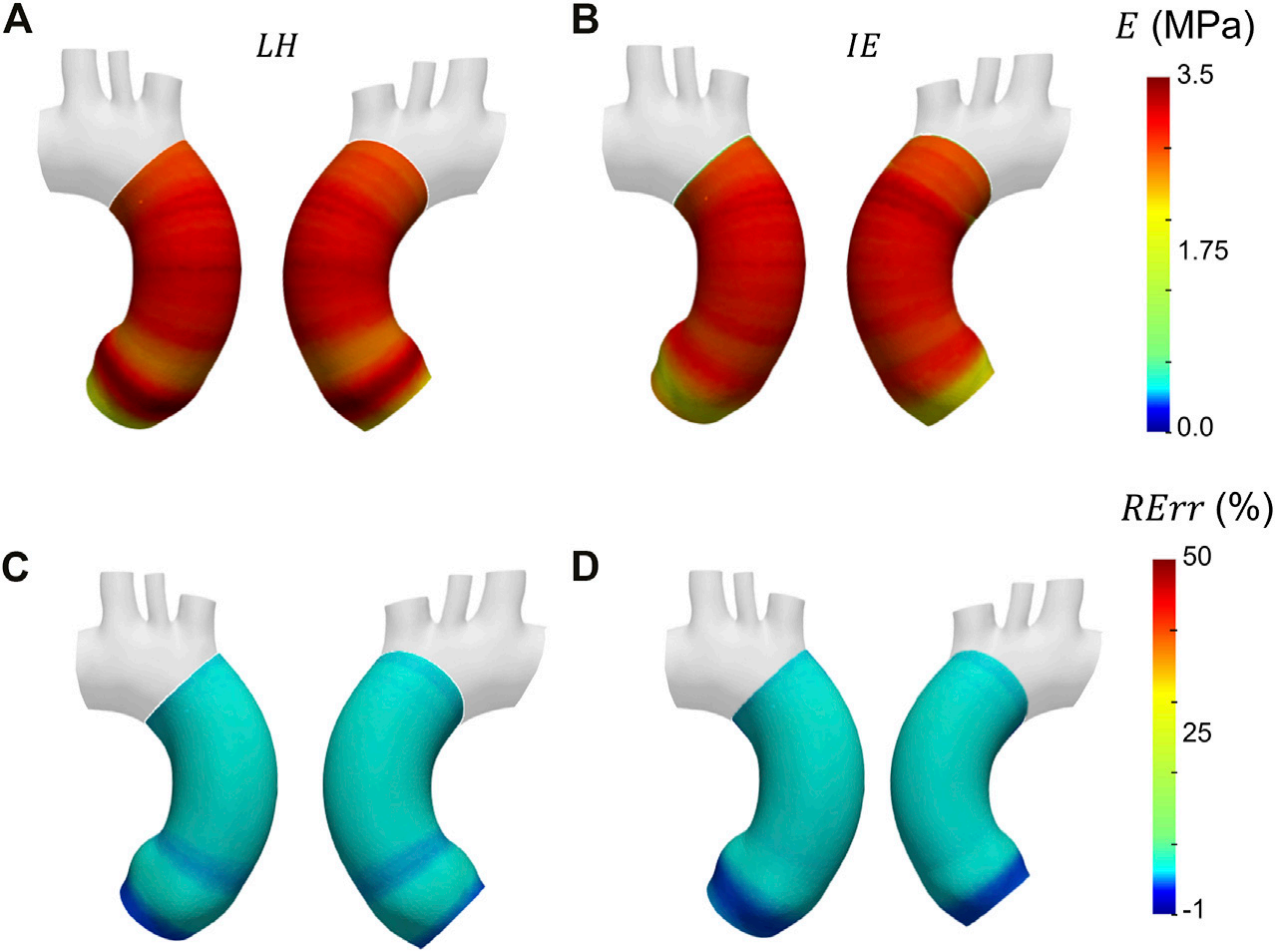
Young’s modulus maps of FE validation for patient-specific geometry with homogeneous distribution with ${\bar{E}}_{\theta }^{H}=$ 3.0 MPa **(A, B)** with corresponding relative errors **(C, D)**. Both Laplace-hypothesis-based $\left(E_{\theta }^{LH}\right)$ **(A, C)** and inverse-engineering-based $\left(E_{\theta }^{IE}\right)$ **(B, D)** results are presented.

**FIGURE 9:**
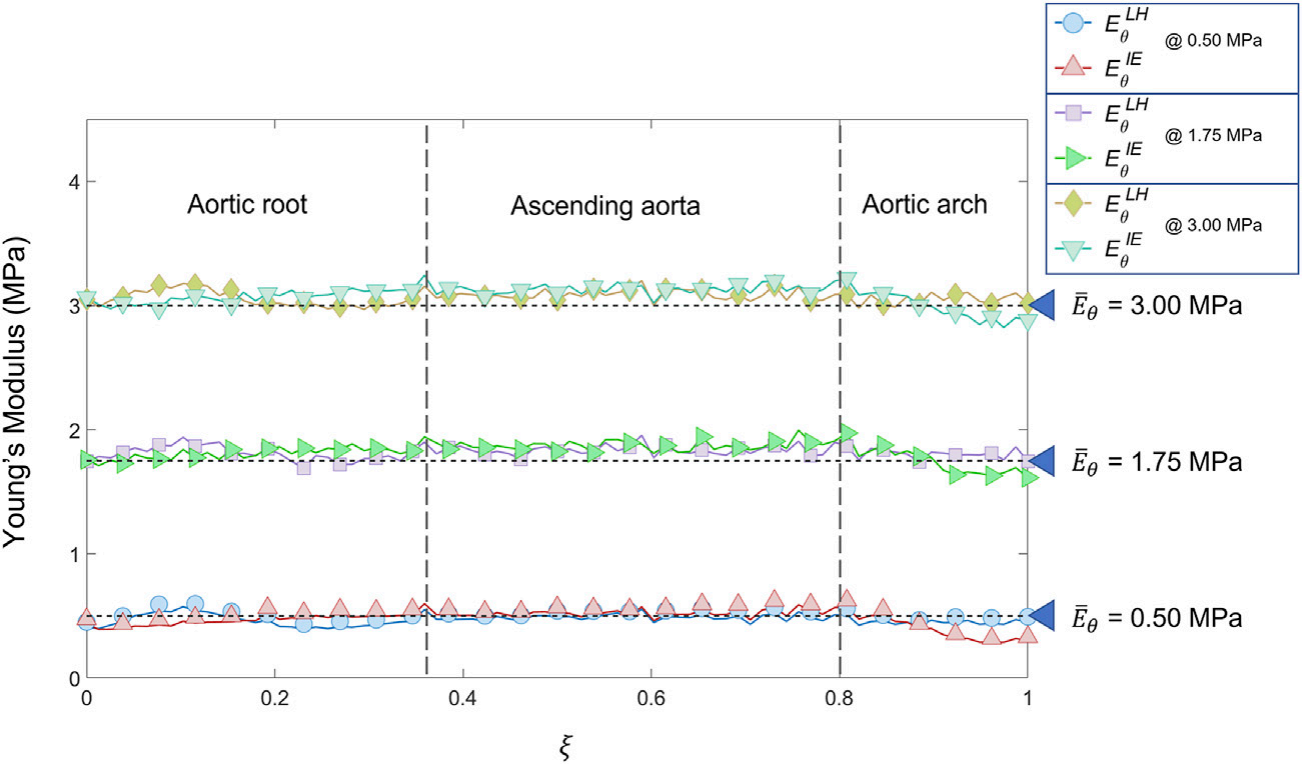
Young’s modulus variation along the centerline coordinate according to both LH and IE methods for the patient-specific homogeneous distributions cases ( ${\bar{E}}_{\theta }^{H}=$ 0.5 MPa, 1.75 MPa, 3.0 MPa) compared with the reference values.

Before presenting the patient-specific validation cases, the negligibility of the curvature was checked by evaluating the curvature effect factor. The geometry revealed an *m* factor suitable for the condition of Eq. 3 (*m* = 0.89). In Figures 6A, B, Figures 7A, B and Figures 8A, B the Young’s modulus maps of the patient-specific validation cases with homogeneous material properties distributions are reported for the imposed values of ${\bar{E}}_{\theta }^{H}=$ 0.5 MPa, 1.75 MPa, 3.0 MPa. The *RErr* maps for are reported as well in Figures 6C, D, 7C, D, 8C, D. The results are presented for both LH and IE method. The distributions along the centerline coordinate for the patient-specific validation cases with homogeneous distributions case are reported in Figure 9. Highlights concerning the location of the centerline coordinate, including aortic root, ascending aorta and aortic arch, are represented in figure as well. The distributions according to both LH and IE methods exhibited approximately a constant trend for all the simulated cases of stiffness. The three reference values of ${\bar{E}}_{\theta }^{H}=$ 0.5 MPa, 1.75 MPa, 3.0 MPa are also reported in the graph with dashed lines. The corresponding RMSPE values are all reported in Table 1, with values ranging from 8.5% and to 10.1%.

**FIGURE 10:**
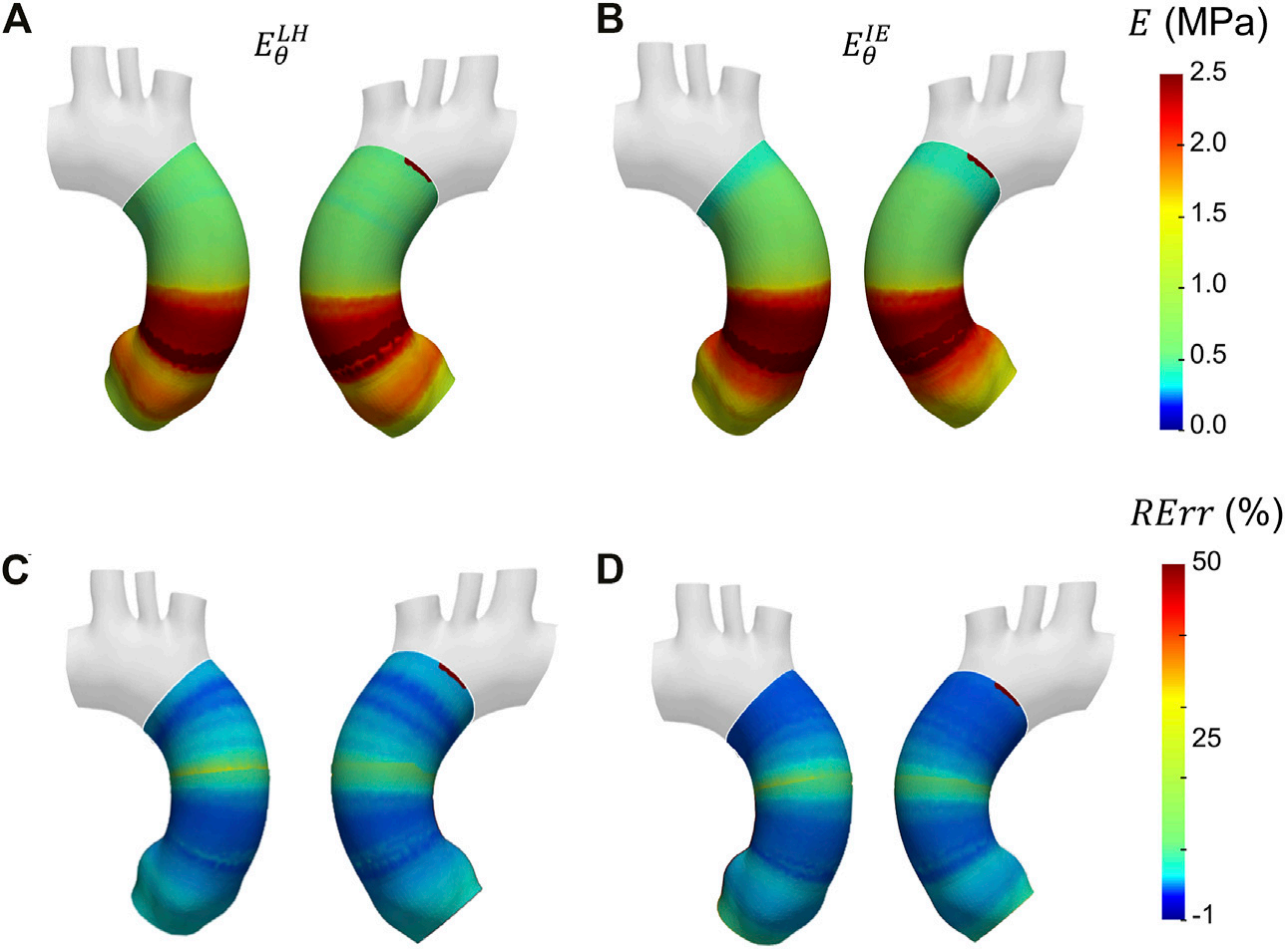
Young’s modulus maps of FE validation for patient-specific geometry with proximal/distal distribution **(A, B)** with corresponding relative errors **(C, D)**. Both Laplace-hypothesis-based $\left(E_{\theta }^{LH}\right)$ **(A, C)** and inverse-engineering-based $\left(E_{\theta }^{IE}\right)$ **(B, D)** results are presented.

**FIGURE 11:**
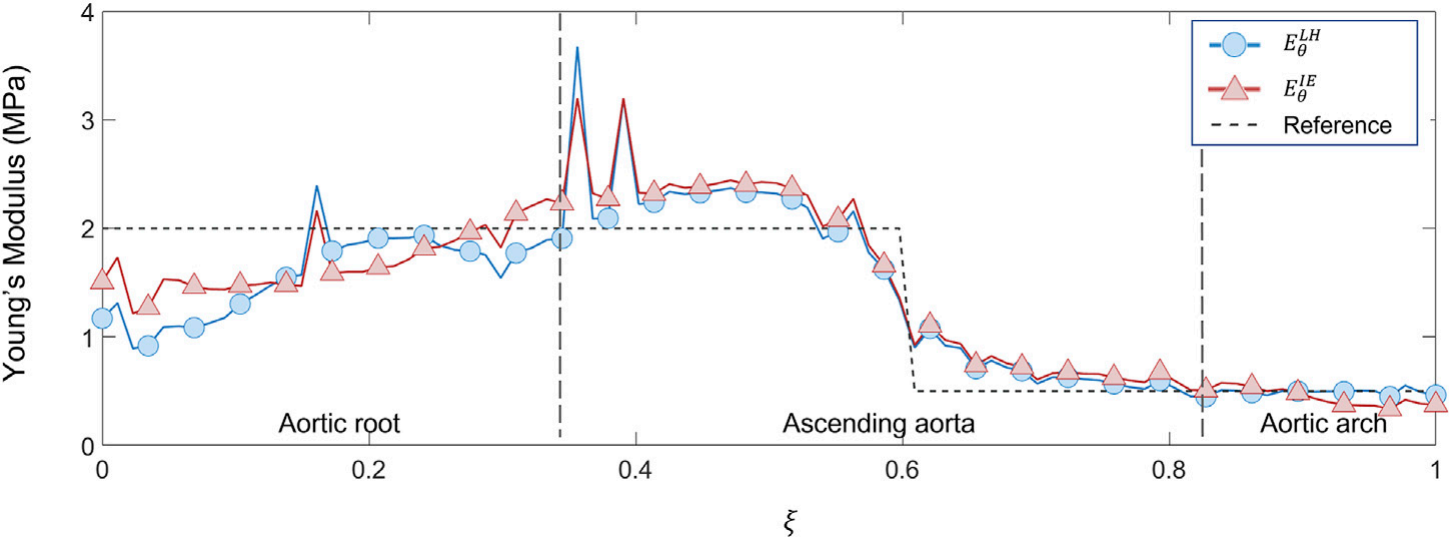
Young’s modulus variation along the centerline coordinate according to both LH and IE methods for proximal/distal distribution case compared with the reference values.

In Figure 10 the Young’s modulus maps of the second validation case with proximal/distal material properties distribution are reported. The different maps of $E_{\theta }^{LH}$ and $E_{\theta }^{IE}$ can be evaluated to determine the performances of both the Laplace-hypothesis-based (Figure 10A) and inverse-engineering-based (Figure 10B) estimation methods in the proximal/distal validation case. The *RErr* maps for are reported as well in Figures 6C, D for both LH and IE methods. The distributions along the centerline coordinate for the proximal/distal validation case are reported in Figure 11. The distributions according to both LH and IE methods exhibited a step-like behavior, with a higher stiffness in the proximal section, as expected. Similarly to the first validation case, the reference values and the highlights concerning the location of the centerline coordinate are reported in the plot. The corresponding RMSPE value are reported in Table 1, with values of 16.3% and 16.1% for the LH and IE methods, respectively.

**FIGURE 12:**
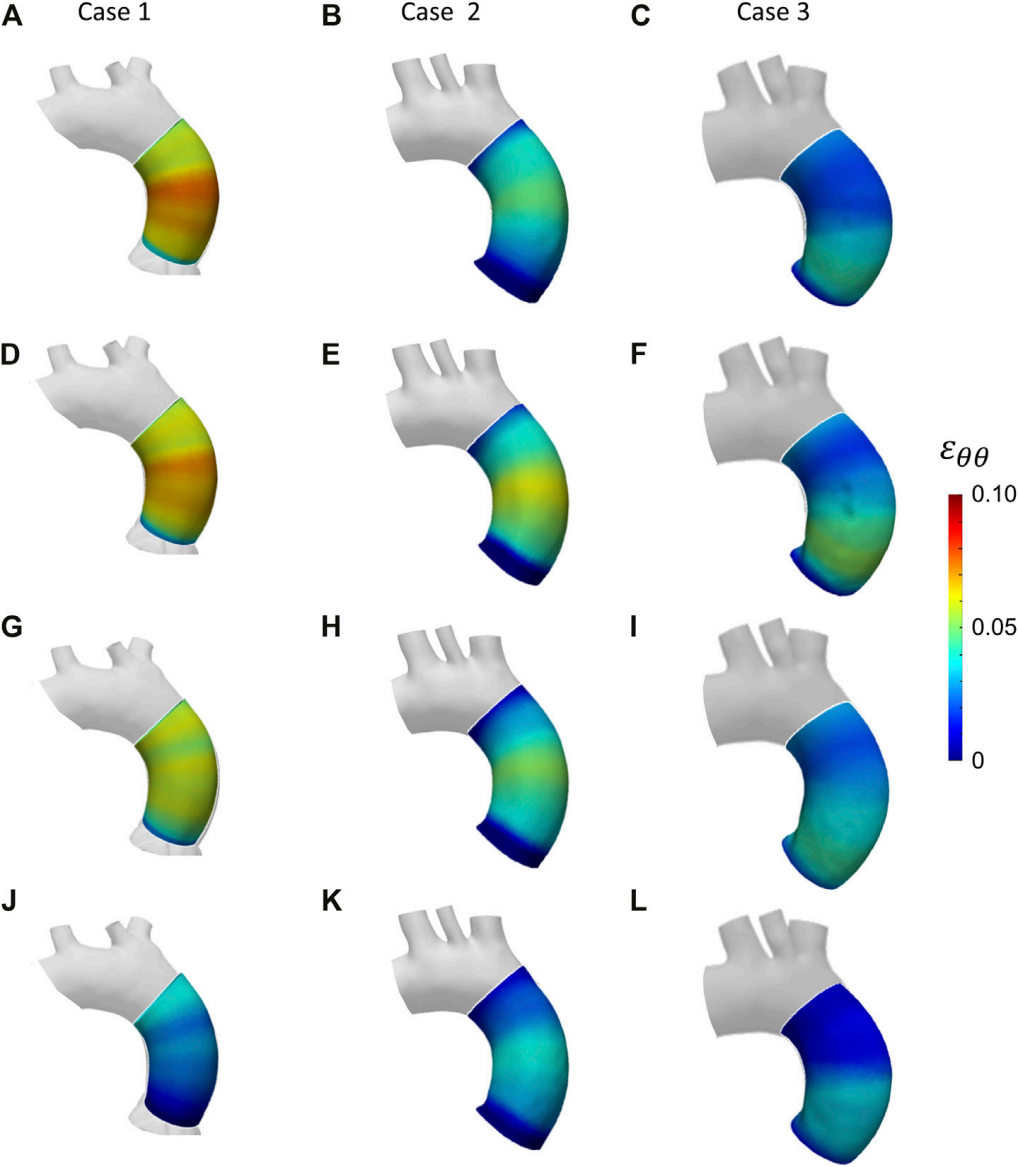
Circumferential strain maps at the four cardiac phases: 20% **(A–C)**; 40% **(D–F)**; 60% **(G–I)**; 80% (**J–L**) from patient-specific Case 1 **(A, D, G, J)**, Case 2 **(B, E, H, K)** and Case 3 (**C, F, I, L**).

The results from the patient-specific cases analyses are then presented. Given the equivalent performances of the estimation methods from the first FE validations, the IE method was chosen for the patient-specific analysis. The values of SNR for each of the patient-specific CT datasets were the following: 35.5 for case 1, 36.1 for case 2 and 37.0 for case 3. The circumferential strain maps at the different cardiac phases from clinical data cases are presented in Figure 12 for all three cases. By inspecting the maximum strain value for each case, it was possible to select the systolic peak phase for each dataset: phase 20% for Case 1, phase 40% for Case 2 and Case 3. The only phase chosen as systolic peak was adopted for the stiffness calculation.

**FIGURE 13:**
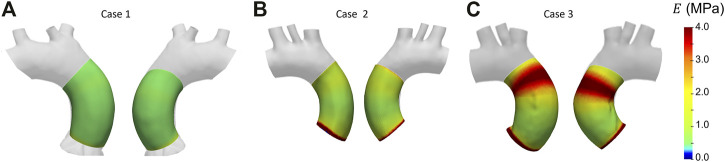
Young’s modulus maps from patient-specific cases: Case 1 **(A)**, Case 2 **(B)** and Case 3 **(C)**.

**FIGURE 14:**
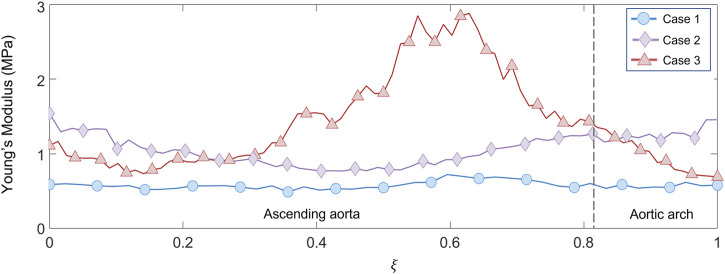
Young’s modulus variation along the centerline coordinate for the three patient-specific cases.

After selecting the systolic peak phase for each case on the basis of the strain, the results in terms of Young’s modulus were calculated. The maps for the different patient-specific cases are reported in Figure 13. The Young’s modulus trends as a function of centerline coordinate are also presented for all the patient-specific cases, as showed in Figure 14. For Case 1 and Case 2, a more homogeneous trend was reported. On the contrary, the behavior of Case 3 appeared to be less homogeneous, as stiffer values were encountered in the ascending aorta in proximity of the aortic arch section.

## 4 Discussion

The results presented in the previous section demonstrate the effectiveness of the proposed technique for the stiffness estimation in the ascending aorta from *in vivo* data. The performances of both Laplace-hypothesis based and Inverse-engineering based approaches have been presented. In particular, the methods were first tested on FE-based validation cases in which homogeneous and heterogeneous Young’s modulus distribution were imposed on both idealized and patient-specific geometries. The proposed approach was successfully implemented also with patient-specific data thanks to shape morphing techniques, revealing the strain and stiffness distribution of three real cases of ascending aortic sections.

The results from the validation on the idealized geometries are presented in Figure 5. From the resulting maps it is possible to assess that the produced errors are always below 10% for all the chosen cases. In particular, it is interesting to notice that for the ideal cylinder there is no substantial difference between the LH and IE estimation methods, as in both cases the results presented correspond to the Laplace theory. In both cases, the cylinder produces negligible errors, below 2% (see also Table 1). By introducing a curvature, the differences between the two methods emerge. In fact, by inspecting the results from the elbow cases, it is evident that the effect of curvature influences the performances of the LH method. This effect is particularly evident for the elbow at 90°, where the curvature factor equals to 0.86, in which the *RErr* values reach a maximum of 7% for the LH method, while the IE method produces maximum errors of 3%. These results demonstrate that the curvature effects the method performances, nevertheless both approaches produced satisfactory estimations of stiffness distribution with errors always remaining below the 10% threshold.

The results obtained in the patient-specific validation cases in which stiffness distribution was imposed as homogeneous are presented in the maps of Figure 6, Figure 7 and Figure 8. From the maps, it is possible to observe that according to both the estimation techniques that the homogeneity of the stiffness was correctly coped for all the imposed values of Young’s modulus. This behavior is confirmed also by the Young’s modulus trend as a function of centerline coordinate from Figure 9. For all the imposed values of Young’s modulus, the same trends have been encountered. In particular, for the LH cases, a wider variation of stiffness values is encountered only within the aortic root section. This behavior can be assumed as a consequence of stress direct dependence on the section radius. This oscillation in the aortic root section is absent instead according to the IE method for all the three values of imposed Young’s modulus. Underestimations are instead encountered within the aortic arch section for the $E_{\theta }^{IE}$ calculation. It is reasonable to assume these underestimations linked with $E_{\theta }^{IE}$ can be caused by imposed boundary conditions at FE simulation level. Nevertheless, it is interesting to highlight that both methods reveal a similar and constant trend within the ascending aorta section, as reported by both the maps of Figure 6, Figure 7 and Figure 8 and also the plots of Figure 9. The similarity of LH and IE performances is also confirmed by the RMSPE values reported in Table 1. In all patient-specific validation cases, the RMSPE percentages were similar, with values around 10%. In particular, a maximum of 10.1% for the LH method with ${\bar{E}}_{\theta }^{H}=$ 0.5 MPa and a minimum of 8.5% for IE method with ${\bar{E}}_{\theta }^{H}=$ 3.0 MPa were experienced. Concerning homogeneous stiffness distributions for the patient-specific validation, these error values make plausible to assume that both LH and IE methods are comparable in terms of performances.

Similar trends were encountered also for the second validation case with heterogeneous distribution. From the maps of Figure 10, the underestimation area in the aortic root zone of the LH method map remains evident, as observed also in the previous validation case. Concerning the IE method, the same underestimation area in the aortic arch area, already observed in the homogeneous validation case, can be highlighted on the heterogeneous validation case. Nevertheless, both LH and IE methods correctly cope the zone distribution of the Young’s moduli in the proximal and distal sections of the ascending aorta. In fact, the transition from the high stiffness ( ${\bar{E}}_{\theta }^{P}$ = 2.0 MPa) area in the proximal section to the low stiffness ( ${\bar{E}}_{\theta }^{D}$ = 0.5 MPa) area in the distal region is correctly marked in both $E_{\theta }^{LH}$ and $E_{\theta }^{IE}$ maps, as showed in Figures 10A, B. The same transitions can also be detected in the graphs of Figure 11, where the Young’s modulus trend according to centerline coordinate is reported. The $E_{\theta }^{LH}$ is oscillating in the aortic root zone, as already observed in the first validation case. Additionally, the $E_{\theta }^{IE}$ underestimation in the aortic arch section remains even in this validation case. It is safe to assume that the underestimation of $E_{\theta }^{IE}$ in the aortic arch section remains linked with the boundary conditions imposed in the FE simulation, as already observed in the first validation case. It is interesting to observe that the estimations remain approximately equivalent, regardless of the method used, within the ascending aorta section. This aspect is confirmed by the presence of the same outliers, encountered in both $E_{\theta }^{LH}$ and $E_{\theta }^{IE}$ trends. Additionally, for both LH and IE case, it was possible to observe the transition along the centerline. The RMSPE are reported in Table 1. The errors are, in fact, equivalent for both LH (16.3%) and IE (16.1%) method. Higher percentage of errors are encountered for the heterogeneous case in comparison with the homogeneous case. This behavior can be motivated by considering that the sudden change in Young’s modulus cannot be completely coped by the strain estimation approach, which necessarily introduces a smoothing action by considering cross sectional planes.

With these validation results, it was possible to assess the performances of the workflow. It was safe to assume that both LH and IE method revealed in general satisfactory performances, with similar errors for both validation cases. The IE method was chosen to proceed with the patient-specific cases. The strain maps at the given cardiac phases were calculated first (Figure 12). The evaluation of strain maps at the different phases allowed for the individuation of the systolic peak for each case, by evaluating the strain maximum. In all cases, a strain below 10% was encountered. The range reported was in accordance with previously observed data calculated on *in vivo* aortic cases (Bell et al. (2014); Satriano et al. (2018); Wilson et al. (2019)). It is also possible to observe heterogeneity from case to case. In particular, Case 1 exhibited the highest values of circumferential strain in comparison with the two other patient-specific cases. In addition, Case 3 revealed an area with high strains at the proximal section of the ascending aorta. The individuation of the systolic peak phase made possible the estimation of the Young’s modulus (Figures 13, 14). The calculated stiffness distributions are in line with the reported strain maps. In fact, while Cases 1 and 2 revealed mainly an homogeneous distribution of Young’s modulus, with average values of 0.6 MPa and 1.0 MPa respectively, Case 3 exhibited a marked heterogeneity, with a stiffer section close to the aortic arch and ranging from 0.7 MPa to 2.9 MPa. It is evident from both the maps of Figure 13 and the trends of Figure 14 that Case 3 revealed an increased stiffness in comparison with the other two cases, with peaks below 3.0 MPa. This phenomenon is in line with the already established connection between arterial stiffness and age, as Case 3 data are associated with the older patient case (Qiu and Onuh (2020)). It was plausible to expect this behavior, also considering the lower strain values encountered from the analysis of the different cardiac phases. Considering all three cases, the estimated values of Young’s modulus were always contained within the 0.5–3.0 MPa range, which is in line with already reported physiological values from the state of the art (Lin et al. (2022); Vignali et al. (2021b)).

Further points of development and limitations of the current workflow can be highlighted. Both the proposed LH and IE do not take into account of inertial loading, as in both cases the assumption of quasi-static solicitations was made. A limitation is given by the reduced number of patient-specific cases tested after validation. To better define the approach outcomes, a wider number of cases will be analyzed and presented in the future. The current method is limited on the estimation of circumferential strain only and it assigns a single value for each centerline-based slice of the aorta. Thus, a limitation of the estimation method in its current state is the impossibility to calculate the inner/outer curvature strain difference on the aorta surface by considering the whole contour length. Moreover, even if the mapping of the surface accounts for the axial displacement of sections, the approach based on the contour length cannot estimate the longitudinal strain. Further developments of the algorithm will allow in the future to estimate the axial deformation of the aorta and they will open up the path for the adoption of more complex constitutive models including anisotropy. Concerning hyperelasticity, it is well established that the aortic tissue has a non-linear mechanical response, given the presence of collagen fibers and the presented method does not account for these phenomena. Nevertheless, it was also established that deformations occurring between diastolic and systolic phase can be assumed as small (Gundiah et al. (2008); Vignali et al. (2021b)). This aspect confirms the hypothesis of linear material behavior assumed in the presented method. To further assess the effectiveness of the proposed method, it would be interesting to test the approach even on a complete aortic geometry, including epiaortic vessels and descending aorta. As an additional point of development, the method can be tested also with CT with ECG-gating with finer time sampling. Concerning the CT image quality, to assess the influence of noise on the presented procedure’s outcomes a full uncertainty quantification process would be required. Nevertheless, the SNR assessment for the processed images of the three patient-specific cases confirmed that the image quality was satisfactory and in line with diagnostic standards (Shen et al. (2015)). In this way the method could have the potential to obtain a more accurate estimation of the systolic peak cardiac phase.

## 5 Conclusion

In summary, the current study presents a new method for local strain and stiffness estimation from *in vivo* ECG-gated CT aortic images, on the basis of mesh-morphing mapping and inverse engineering methods. The method was first validated on two test cases, obtained from FE simulations of aortic structures with different material properties local distributions. After a successful validation, the method was applied on three patient-specific aorta cases. The results demonstrated the successful obtainment of a regional *in vivo* characterization of patient-specific aortas in terms of deformations and stiffness.

## Data Availability

The raw data supporting the conclusions of this article will be made available by the authors, on request from the corresponding author, without undue reservation.
